# Congenital Malignant Ectomesenchymoma Presenting as a Neck Mass in a Newborn

**DOI:** 10.3390/children12040480

**Published:** 2025-04-08

**Authors:** Ianna S. C. Blanchard, Ravi C. Bhavsar, Ashley M. Olszewski, Nathan R. Shelman, John A. D’Orazio, Prasad Bhandary, Thitinart Sithisarn

**Affiliations:** 1 Department of Pediatrics—Neonatology, University of Kentucky, Lexington, KY 40506, USA; ravi.bhavsar@uky.edu (R.C.B.); aolszewski@uky.edu (A.M.O.); prasad.bhandary@uky.edu (P.B.); tsith2@uky.edu (T.S.); 2 Department of Pathology, University of Kentucky, Lexington, KY 40506, USA; nathan.shelman@uky.edu; 3 Department of Pediatrics—Hematology/Oncology, University of Kentucky, Lexington, KY 40506, USA; jdorazio@uky.edu

**Keywords:** newborn, neonatology, malignant ectomesenchymoma, congenital neck mass, soft tissue neoplasm

## Abstract

**Background**: Malignant Ectomesenchymoma (MEM) is a rare, aggressive soft tissue neoplasm with both neuroectodermal and mesenchymal differentiation. Congenital cases are extremely uncommon, posing significant diagnostic and therapeutic challenges. **Case Presentation**: We report a case of a full-term male neonate presenting with a large congenital neck mass and respiratory distress at birth. Imaging revealed a lobulated, heterogeneously enhancing mass in the left submandibular region with a mass effect on the airway. Open biopsy and gross resection on day six of life confirmed MEM with rhabdomyoblastic and neuroectodermal differentiation. Post-surgical staging classified the tumor as Stage I, Clinical Group II. Despite initial chemotherapy with Vincristine, Actinomycin, and Cyclophosphamide (VAC), tumor recurrence was detected at week nine of chemotherapy, necessitating a transition to Vincristine, Irinotecan, and Temozolomide (VIT). **Discussion**: MEM is an extremely rare neoplasm in infants, particularly in congenital presentations. Diagnosis is challenging due to its mixed histopathological features and broad differential diagnosis, including rhabdomyosarcoma, fibrosarcoma, lymphangioma, and neuroblastoma. Management typically involves multimodal therapy, with surgical resection being the mainstay of treatment. Chemotherapy is often tailored to the tumor’s most aggressive component, though standardized treatment protocols remain undefined. **Conclusions:** This case highlights the importance of early recognition and a multidisciplinary approach in managing congenital MEM, as a differential diagnosis of soft tissue masses in infants, particularly in the head and neck region.

## 1. Introduction

Malignant Ectomesenchymoma (MEM) is an exceedingly rare soft tissue neoplasm. MEM is considered a skeletal muscle malignancy [1], presumed to originate from migratory neural crest cells, but the exact etiology is unclear [2,3,4]. MEM is biphasic, characterized by neuroectodermal (with components of varying degrees of maturation) and mesenchymal elements (often featuring predominantly rhabdomyoblastic differentiation) [3,4].

MEM tumors may develop at any site within the soft tissue or central nervous system. Most commonly, it presents in the soft tissues within the abdomen, pelvis, or external genitalia; and less commonly, within the head–neck regions or mediastinum [3]. Most cases are diagnosed in infants or children under 2 years of age; congenital presentations are exceedingly uncommon, with less than 100 cases reported in the literature [3,4]. MEM poses significant diagnostic and therapeutic challenges due to its mixed nature and rarity. This case report describes a newborn with congenital MEM of the neck admitted to the Neonatal Intensive Care Unit (NICU).

## 2. Case Presentation

A caucasian full-term, large for-gestational-age male neonate, born via cesarean section at 39 weeks gestation, was admitted to a level-four NICU in Kentucky for a large mass on the left lateral aspect of his neck, and respiratory distress. The pregnancy was complicated by gestational diabetes, maternal history of incompletely treated latent tuberculosis, and a vascular malformation on a lower extremity. Additionally, there is a non-contributory family history of head and neck cancer. Notably, routine prenatal ultrasound was unremarkable at 38.0 weeks.

**Figure 1:**
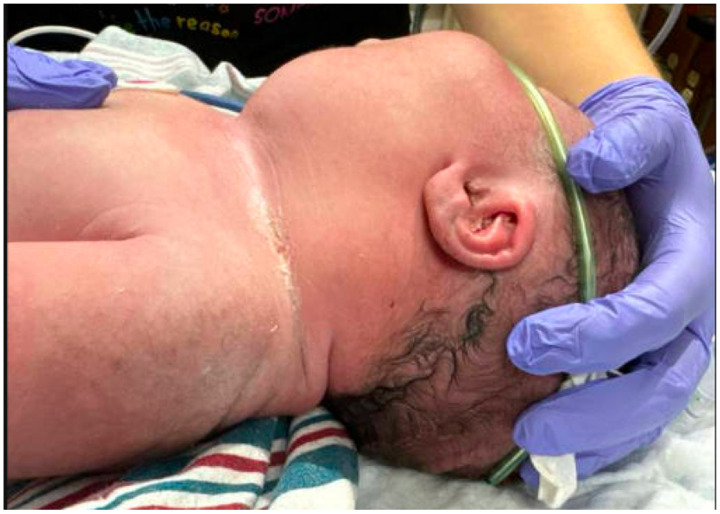
Left lateral aspect of infant’s neck on day of life 0.

At birth, the newborn physical examination revealed a large, non-tender firm submandibular mass, measuring approximately 5 cm × 2 cm on the left neck, below the ear. The mass was mobile and well demarcated, without overlying skin changes (Figure 1). Stridulous at rest, the infant was most comfortable laying on his left side but was otherwise well appearing. He was maintained on continuous positive airway pressure (CPAP) and supplemental oxygen.

Initial laboratory investigations demonstrated no significant anemia, leukocytosis, or thrombocytopenia and normal hepatic function tests. Homovanillic acid (HVA), vanillylmandelic acid (VMA) measurements, and coagulation panel were within normal limits. Evaluation by pediatric otolaryngology showed a patent airway with bulging of the left posterior pharyngeal wall, demonstrating a mass effect on the airway.

**Figure 2:**
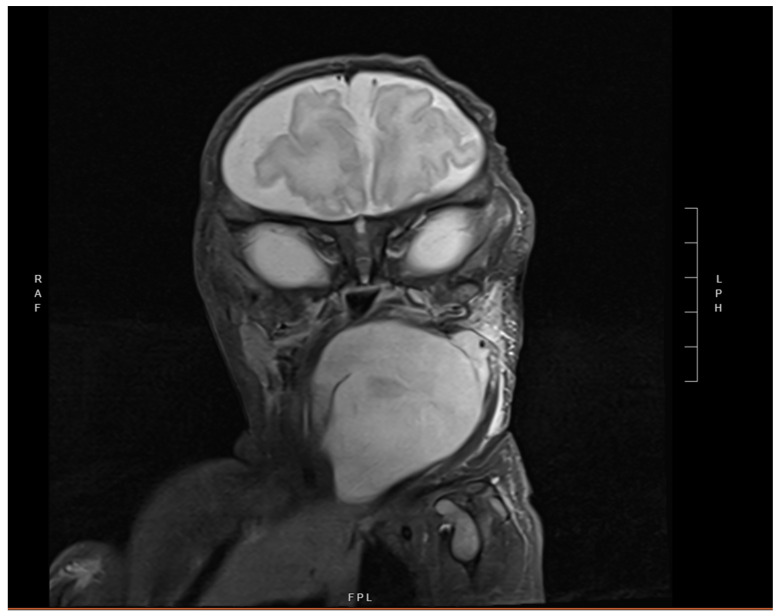
Coronal short tau inversion recovery (STIR) MRI image demonstrating 2.2 × 5.2 × 5.1 cm neck mass in left parapharyngeal space.

Neck Ultrasound demonstrated a well-circumscribed 7.1 × 4.1 × 4.7 cm hyperechoic lesion in the submandibular region with mild internal color Doppler flow. A contrast-enhanced magnetic resonance imaging (MRI) revealed a large, lobulated, multi-spatial, heterogeneously enhancing, 2.2 × 5.2 × 5.1 cm mass centered in the left neck and extending medially. Significant mass effect with enhancement was seen on adjacent structures including the oropharynx and larynx with soft tissue stranding, and edema was present (Figure 2). Head MRI showed an incidental right-sided prominence of the extra-axial space.

**Figure 3:**
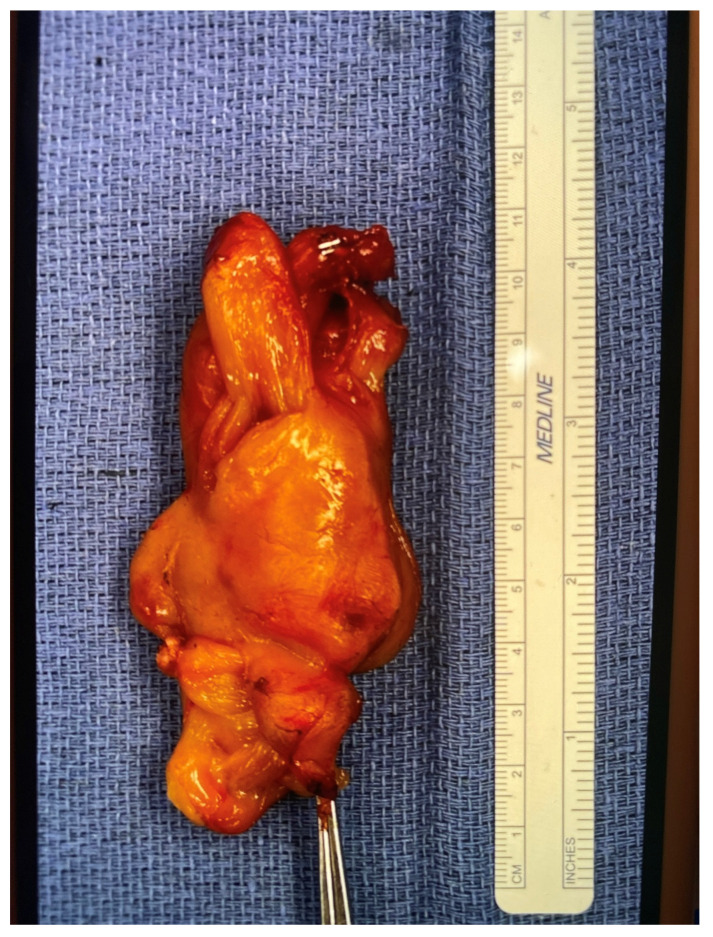
Gross specimen of a resected neck mass.

**Figure 4:**
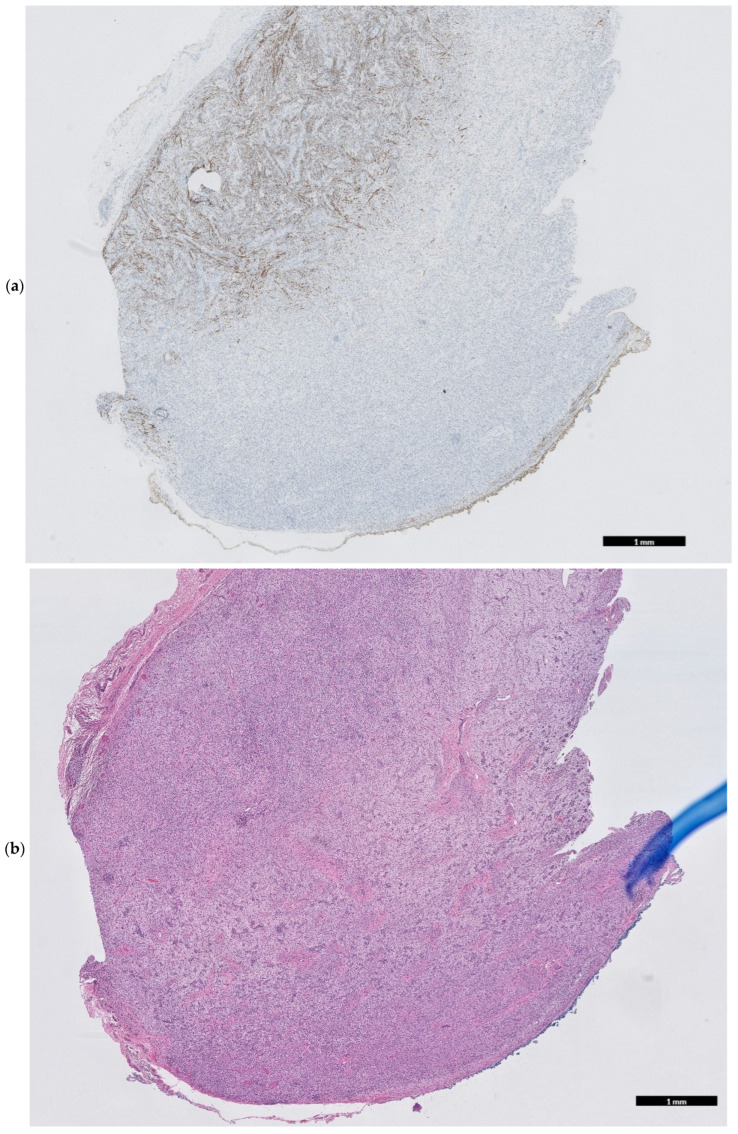

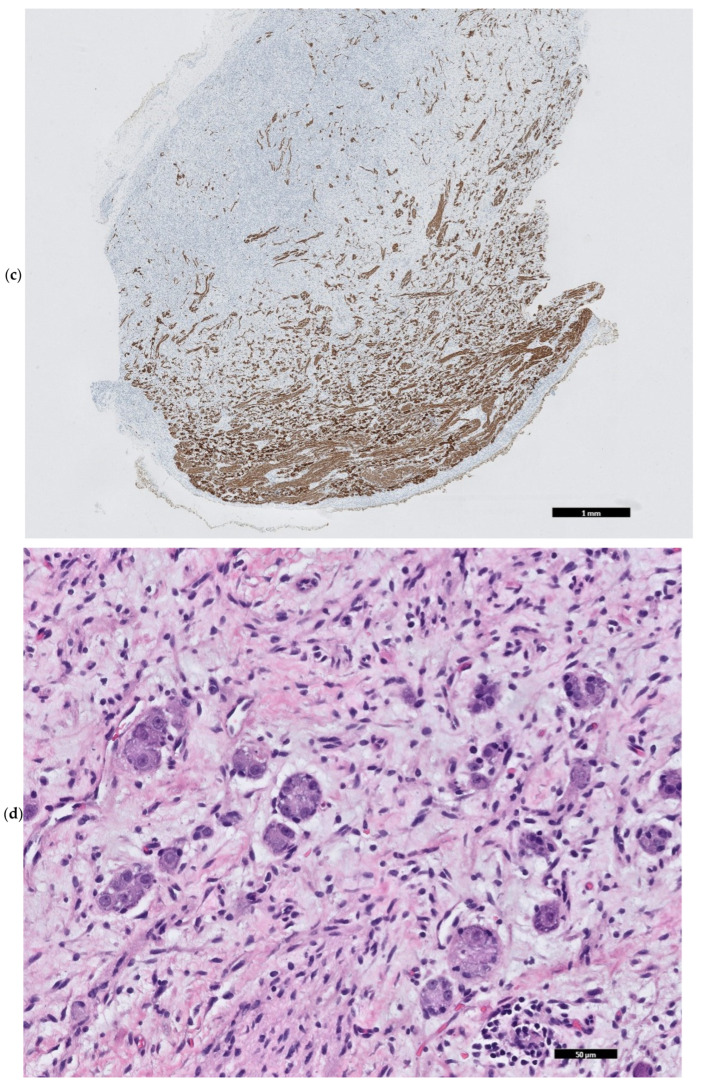

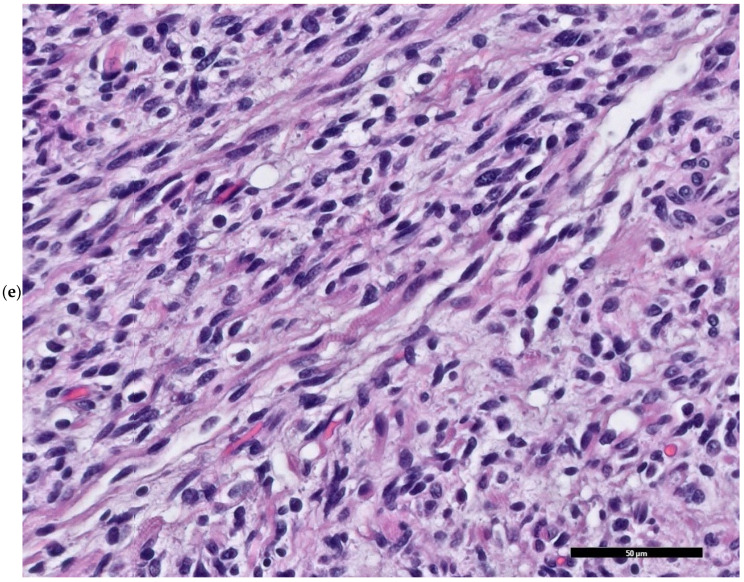
(**a**) Hematoxylin and Eosin (H&E) slide of neck MEM—neuroectodermal component (bottom) vs. RMS component (top left) (Desmin IHC, 10×). (**b**) H&E slide of neck MEM—neuroectodermal component (bottom) vs. RMS component (top left) (H&E, 10×). (**c**) H&E slide of neck MEM—Neuroectodermal component (bottom) vs. RMS component (top left) (Synaptophysin IHC, 10×). (**d**) H&E slide of neck MEM—neuroectodermal component (loose spindly stroma with clustered ganglion cells) (H&E, 200×). (**e**) H&E slide of neck MEM—rhabdomyosarcoma component. Spindled cells with wispy pink muscle differentiation including striations (central) (H&E, 200×).

Open biopsy and gross resection of the mass were performed on day six of life Grossly, the tumor was lobulated, composed of pink-tan tissue, and non-adherent to surrounding tissues (Figure 3). Histological examination showed spindled, stellate, and ovoid cells, with tumor cell cross striations, occasional strap cells, and varied tumor cellularity (Figure 4a–e). There were focal features of neuroectodermal differentiation including Schwannian stroma and maturing ganglion cells. Immunohistochemical staining was positive for S100, synaptophysin, and SOX-10 in areas of neuroectodermal differentiation, while the majority of the spindled cell tumor mass was immunoreactive for desmin, myogenin, and SMA indicative of rhabdomyoblastic differentiation; overall findings were consistent with a diagnosis of malignant ectomesenchymoma (MEM). The Ki-67 proliferative index approached 60–70% in cellular foci. Genetic testing revealed no clinically significant variants of pediatric inherited cancer risk genes. A Comprehensive Cancer Next Generation Sequencing (NGS) Panel (Table A1) molecular profiling revealed loss of *PTCH-1* in the tumor. The patient subsequently tested negative for germline variants of *PTCH-1*, alterations of which have been reported in cases of nevoid basal cell carcinoma syndrome (NBCCS), fetal rhabdomyoma, as well as rhabdomyosarcoma.

**Figure 5:**
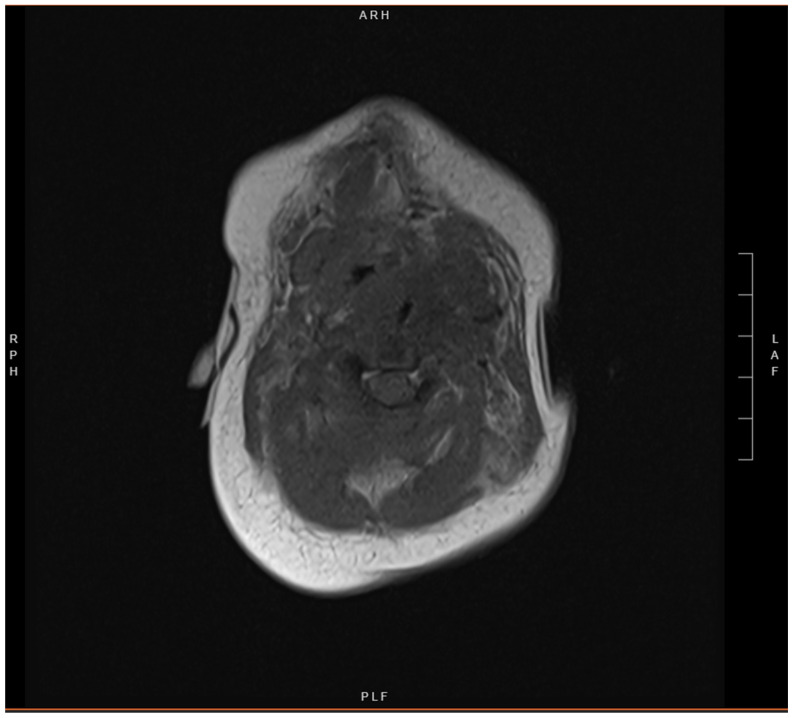
Axial short tau inversion recovery (STIR) MRI image demonstrating post-surgical changes at the level of the larynx from interval resection.

Staging work-up included a post-resection positron emission tomography (PET) scan, computed tomography (CT) chest, and bone marrow biopsy, all of which showed no evidence of metastatic disease. Post-surgical MRI documented the removal of most of the mass; however, there was still some mass effect on the aerodigestive tract, including residual mass effect upon the airway (Figure 5). The final staging classified the tumor as stage I, clinical group II. Multidisciplinary care coordination involved neonatology, pediatric otolaryngology, pediatric oncology, and pediatric surgery.

**Figure 6:**
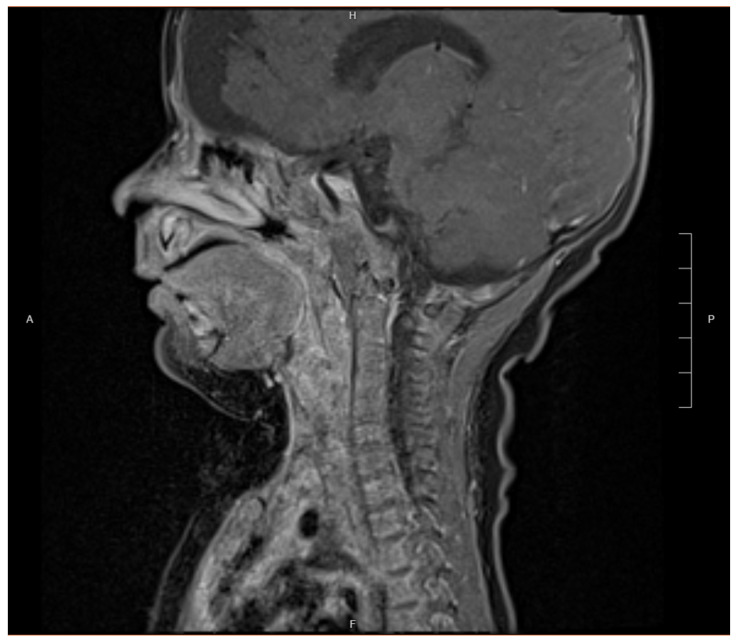
Dixon sagittal post-MRI image demonstrating enhancing mass along left prevertebral soft tissue in retropharyngeal space after resection and nine cycles of VAC chemotherapy.

Following tumor resection, chemotherapy with Vincristine, Actinomycin, and Cyclophosphamide (VAC) was initiated. After nine weeks of chemotherapy, restaging imaging demonstrated a new enhancing 0.8 × 1.5 × 2.1 cm mass in the left retropharyngeal space, causing mass effect on the surrounding structures, and nodal involvement, concerning residual or recurrent disease (Figure 6). Biopsy findings were consistent with residual disease or recurrence of MEM, and the patient was transitioned to a regimen of vincristine, irinotecan, and temozolomide (VIT).

## 3. Discussion

Malignant Ectomesenchymoma (MEM) is an extremely rare heterogeneous soft tissue neoplasm, comprising both neuroectodermal and mesenchymal elements. MEM is considered a skeletal muscle malignancy [1]. The exact etiology is unclear, but it is presumed to arise from migratory neural crest cells [2,3,4]. The mesenchymal component features predominantly rhabdomyoblastic differentiation, such as spindle cells and primitive round cell patterns. The accompanying neuroectodermal component may have varying degrees of maturation, presenting as neuroblastoma, ganglioneuroblastoma, ganglioneuroma, or ganglion cells [3,4].

MEM tumors may develop at any site within the soft tissue or central nervous system. Most commonly, it presents in the soft tissues within the abdomen, pelvis, or external genitalia; and less commonly, within the head–neck regions or mediastinum [3]. Most cases are diagnosed in infants or children under 2 years of age [4]; congenital presentations are exceedingly uncommon, with less than 100 cases globally reported in the literature [3,5]. MEM poses significant diagnostic and therapeutic challenges due to its mixed nature and rarity. This case report describes a newborn with congenital MEM of the neck admitted to a Neonatal Intensive Care Unit (NICU). To our knowledge, this is the first case of MEM that was clinically present at birth and located within the neck. Congenital presentation of MEM is particularly uncommon, with only a few cases reported in the literature. The rarity of MEM, particularly in the congenital form, poses challenges to diagnosis, treatment, and understanding of its pathophysiology.

Most MEM in infants arise from pelvic, retroperitoneal, and urogenital sites, followed by head and neck regions, as seen in our patient, then intracranial and extremity sites, manifesting as painless, rapidly enlarging masses [3]. Some studies suggest MEM shows a male predominance [3,5] and is most often observed in the first two years of life. The pathogenesis of MEM is unclear, though it is believed to arise from undifferentiated neural crest cells, which differentiate into both ectodermal (epithelial) and mesodermal (mesenchymal) tissues [6,7]. Some literature suggests mutations in P53, and cell cycle control pathway regulators could be the drivers of tumorigenesis. Clinical presentation in infants is often nonspecific which can make diagnosis challenging. Most cases present rapidly enlarging masses in the pelvis, abdomen, external genitalia, and head and neck region [4,5,6]. Symptoms can vary depending on the location and size of the tumor, visible swelling, signs of local invasion, or systemic symptoms. Masses in the head and neck region may manifest with symptoms of difficulty breathing or feeding due to obstruction of the aerodigestive tract. The differential diagnosis for congenital neck masses in neonates includes vascular or lymphatic malformations, thyroglossal duct cysts, branchial cleft cysts, or other soft tissue tumors such as congenital fibrosarcoma, hemangiomas, lymphangiomas, and neuroblastomas [5].

Due to the aggressive nature of MEM, a rapid increase in size is often noted during the first few months of life. Diagnosis relies on the combination of imaging studies, histopathological evaluation, and immunohistochemical markers. Imaging ultrasound, CT, and MRI aid in assessing the extent of tumor involvement. Our patient underwent a contrast-enhanced MRI of the head and neck, which revealed a large, enhancing mass exhibiting a mass effect on surrounding tissue, suggestive of a neoplastic lesion. The differential diagnosis for a congenital mass in the neonate is broad and includes conditions such as sarcomas (such as rhabdomyosarcoma (RMS), neuroblastoma, teratoma, benign/malignant triton tumor, and ganglioneuroma), which may be present similarly. MEM has several analogous characteristics with RMS, including anatomic distribution, demographic, and histologic features [3]. The biphasic pattern of MEM makes the tumor histologically distinctive, as it demonstrates both glandular epithelial differentiation and mesenchymal features. Most cases demonstrate rhabdomyoblastic differentiation [7,8], such as spindle cells (which may resemble cartilage or fibrous tissue), chondroid differentiation, and neuroblastic features [6]. The neuroectodermal components present in MEM can encompass a range of neuroblastic phenotypes, including scattered ganglion cells, ganglioneuroma, ganglioneuroblastoma, and neuroblastoma [5,9]. Associated immunohistochemical markers include neural markers such as S100 protein, synaptophysin, and mesenchymal markers such as desmin, and myogenin [5,6,7]. In our case, the tumor exhibited a predominantly rhabdomyoblastic spindle cell proliferation as well as focal clustered ganglion cells enmeshed in mature Schwannian stroma, findings consistent with the diagnosis of MEM.

Management of MEM is challenging due to its aggressive nature and the heterogeneity of the tumor, which complicates both diagnosis and treatment. Adequate biopsy specimen is essential for accurate diagnosis and regimen selection, based on the highly variable biphasic nature of MEM, an inadequate sample may be histologically indistinguishable from RMS or the neuroectodermal components [9]. Consensus supports a multimodal approach to treatment, including surgical resection, chemotherapy, and radiation therapy [3,5,8]. Complete surgical excision is the primary treatment modality when feasible for MEM, offering an excellent prognosis if complete resection is achieved [5,10]. This is especially true for tumors that are benign or low-grade malignant. If the tumor is unable to be resected due to size, invasion of surrounding tissue, or metastasis to distant sites, the prognosis is poorer. Adjuvant chemotherapy and radiation therapy are indicated when the tumor invades critical structures such as the brainstem, spinal cord, or large vessels, or it has metastasized to other sites. No standard chemotherapy protocol for infants exists. Instead, chemotherapy regimens are tailored for individual cases and typically based on the “most aggressive component” of the mesenchymal elements, which is usually the rhabdomyosarcoma [5,8,9]. Adjuvant radiotherapy can be considered but there is limited data on the role of radiotherapy in MEM, with potentially significant long-term side effects on growth and development [5].

Close follow-up is necessary to monitor for recurrence, although recurrence rates are typically low when clear surgical margins are obtained. Gross total resection was achieved in our case with a negative post-resection PET scan, with recurrence within three months. The prognosis for neonates with MEM depends on several factors, including tumor size, location, extent of local invasion, and completeness of resection [3,5,8]. Pellegrino et al. found that “MEMs have the same prognosis as other pediatric chemotherapy sensitive soft tissue sarcomas, with 82% (14/17) of children affected by MEM surviving with no evidence of disease (NED) following multimodality treatment approach” [3]. Further characterization of tumorigenesis and molecular analysis may aid in establishing novel therapies and treatment protocols [4].

This case report has several limitations. As a single-case study, the findings are not directly generalizable. The rarity of MEM limits the availability of comparative cases in the literature, making it challenging to draw definitive conclusions about disease patterns, treatment efficacy, or prognosis. Furthermore, the lack of long-term follow-up data available for the case prevents us from assessing the patient’s long-term response to treatment or the possibility of recurrence. Another limitation is the absence of further comprehensive molecular and genetic analyses, which could have provided further insights into the tumor’s pathogenesis and potential therapeutic targets. Lastly, due to the limited literature on MEM, our discussion is based on the limited number of previously reported cases.

## 4. Conclusions

Congenital ectomesenchymoma is an extraordinarily rare aggressive tumor that presents significant diagnostic and management challenges in neonates. This case highlights the importance of early recognition, comprehensive imaging, and histochemical analysis of MEM, as a differential diagnosis of soft tissue masses in infants, particularly in the head and neck region.

## Data Availability

The original contributions presented in this study are included in the article. Further inquiries can be directed to the corresponding author.

## Abbreviations

The following abbreviations are used in this manuscript:

MEM	Malignant Ectomesenchymoma
NICU	Neonatal Intensive Care Unit
CPAP	Continuous Positive Airway Pressure
HVA	Homovanillic Acid
VMA	Vanillylmandelic Acid
MRI	Magnetic Resonance Imaging
PET	Positron Emission Tomography
CT	Computed Tomography
VAC	Vincristine, Actinomycin, and Cyclophosphamide
VIT	Vincristine, Irinotecan, and Temozolomide
H&E	Hematoxylin and Eosin
STIR	Short Tau Inversion Recovery
RMS	Rhabdomyosarcoma

## Appendix A

**Table A1:** (**a**) Comprehensive Cancer Next Generation Sequencing (NGS) Panel DNA gene list (543 genes). Only CNV analysis or ^—Internal Tandem Duplication (ITD) interrogated; $—Exon 1 region not entirely sequenced [11,12]. (**b**) Comprehensive Cancer Next Generation Sequencing (NGS) Panel RNA gene list (183 genes) * CTNNB1 exon—level (large) deletions, ^—EGFRvIII, and $—MET exon 14 skipping variant interrogated in addition to gene fusion [12].

(**a**)									
*ABL1*	*ABL2*	*ABRAXAS1 **	*ACVR1*	*ACVR1B **	*ADGRA2 **	*AIP*	*AKT1*	*AKT2*	*AKT3*
*ALK*	*AMER1*	*APC*	*AR*	*ARAF*	*ARFRP1 **	*ARID1A*	*ARID1B $*	*ARID2*	*ASXL1*
*ATM*	*ATR*	*ATRX*	*AURKA*	*AURKB*	*AXIN1*	*AXIN2*	*AXL*	*B2M*	*BAP1*
*BARD1*	*BCL10 **	*BCL11B **	*BCL2*	*BCL2L1 **	*BCL2L2 **	*BCL6*	*BCL7A*	*BCOR ^*	*BCORL1*
*BIRC3*	*BLM*	*BMPR1A*	*BRAF*	*BRCA1*	*BRCA2*	*BRCC3 **	*BRD3 **	*BRD4*	*BRINP3 **
*BRIP1*	*BTG1*	*BTK*	*BUB1B **	*C19MC*	*CALR*	*CARD11*	*CASP8 **	*CBFB*	*CBL*
*CBLB*	*CBLC*	*CCN6*	*CCND1*	*CCND2*	*CCND3*	*CCNE1*	*CD19 **	*CD274*	*CD28 **
*CD40 **	*CD58*	*CD79A*	*CD79B*	*CDC73*	*CDH1*	*CDK12*	*CDK4*	*CDK6*	*CDK8*
*CDKN1A*	*CDKN1B*	*CDKN1C*	*CDKN2A*	*CDKN2B*	*CDKN2C*	*CEBPA*	*CHD2*	*CHD4*	*CHEK1*
*CHEK2*	*CIC*	*CIITA **	*COL1A1*	*CREBBP*	*CRKL*	*CRLF2*	*CSF1R*	*CSF3R*	*CSNK1A1 **
*CTCF*	*CTLA4 **	*CTNNA1 **	*CTNNB1*	*CUL3*	*CUX1*	*CXCR4*	*CYLD*	*DAXX*	*DDR2*
*DDX3X*	*DDX41*	*DGCR8*	*DICER1*	*DIS3L2*	*DKC1*	*DNM2*	*DNMT3A*	*DOT1L*	*DROSHA*
*EBF1*	*EED*	*EFNB2 **	*EGFR*	*EGR2 **	*EIF1AX*	*ELOC*	*EMSY **	*EP300*	*EPCAM*
*EPHA3*	*EPHA5*	*EPHA7 **	*EPHB1*	*EPHB4 **	*ERBB2*	*ERBB3*	*ERBB4*	*ERCC1 **	*ERCC2*
*ERCC3 **	*ERCC4 **	*ERCC5 **	*ERG **	*ERRFI1 **	*ESR1*	*ETNK1*	*ETS1 **	*ETV1 **	*ETV4 **
*ETV5 **	*ETV6*	*EWSR1 **	*EXT1*	*EXT2*	*EZH2*	*FANCA*	*FANCB*	*FANCC*	*FANCD2 **
*FANCE **	*FANCF **	*FANCG **	*FANCI*	*FANCL **	*FANCM **	*FAS*	*FAT1*	*FAT3 **	*FBXO11*
*FBXW7*	*FGF10 **	*FGF14 **	*FGF19*	*FGF23 **	*FGF3*	*FGF4*	*FGF6 **	*FGFR1*	*FGFR2*
*FGFR3*	*FGFR4*	*FH*	*FLCN*	*FLT1*	*FLT3 ^*	*FLT4*	*FOXA1*	*FOXL2*	*FOXO1 **
*FOXP1*	*FOXR2 **	*FRK*	*FRS2 **	*FUBP1*	*FUS **	*GABRA6 **	*GATA1*	*GATA2*	*GATA3*
*GATA4 **	*GATA6 **	*GEN1 **	*GID4 **	*GLI1*	*GLI2*	*GNA11*	*GNA13*	*GNA14*	*GNAQ*
*GNAS*	*GNB1*	*GPC3 **	*GREM1 **	*GRIN2A*	*GRM3 **	*GSK3B*	*H1-4*	*H3-3A*	*H3-3B*
*H3C2*	*H3C3*	*HDAC4*	*HDAC9*	*HGF*	*HIF1A*	*HMGA2 **	*HNF1A*	*HOXB13 **	*HRAS*
*HSD3B1*	*HSP90AA1 **	*ID3*	*IDH1*	*IDH2*	*IGF1R*	*IGF2 **	*IKBKE*	*IKZF1*	*IKZF2*
*IKZF3*	*IL2RG **	*IL6ST*	*IL7R*	*INHBA **	*INPP4B*	*IRF2*	*IRF4*	*IRF8 **	*IRS2 **
*ITK **	*ITPKB **	*JAK1*	*JAK2*	*JAK3*	*JAZF1 **	*JUN **	*KAT6A **	*KBTBD4*	*KCNJ5*
*KDM4C*	*KDM5A*	*KDM5C*	*KDM6A*	*KDR*	*KEAP1*	*KEL **	*KIF1B **	*KIF5B **	*KIT*
*KLF2*	*KLF4*	*KLHL6 **	*KMT2A*	*KMT2B*	*KMT2C*	*KMT2D*	*KRAS*	*LEF1*	*LIFR **
*LMO1*	*LRP1B **	*LYN **	*LYST **	*LZTR1*	*MAGI2 **	*MAML2 **	*MAP2K1*	*MAP2K2*	*MAP2K4*
*MAP3K1*	*MAP3K14*	*MAP3K3 **	*MAP3K7 **	*MAPK1*	*MAPK3*	*MAX*	*MCL1*	*MDM2*	*MDM4*
*MECOM*	*MED12*	*MEF2B*	*MEN1*	*MET*	*MITF*	*MLH1*	*MLH3*	*MLLT1*	*MN1*
*MPL*	*MRE11*	*MSH2*	*MSH3*	*MSH6*	*MTOR*	*MUTYH*	*MYB*	*MYBL1*	*MYC*
*MYCL **	*MYCN*	*MYD88*	*MYH9 **	*MYOD1*	*NBN*	*NCOA2 **	*NCOR1*	*NCOR2*	*NF1*
*NF2*	*NFE2*	*NFE2L2*	*NFKBIA*	*NFKBIE*	*NKX2-1*	*NOTCH1*	*NOTCH2*	*NOTCH3*	*NPM1*
*NRAS*	*NSD1*	*NSD2*	*NT5C2*	*NTHL1*	*NTRK1*	*NTRK2*	*NTRK3*	*NUP214 **	*NUP93 **
*NUTM1 **	*OTX2*	*PAK3*	*PALB2*	*PAX3 **	*PAX5*	*PAX7 **	*PAX8 **	*PBRM1*	*PDCD1*
*PDCD1LG2 **	*PDGFB **	*PDGFRA*	*PDGFRB*	*PDK1 **	*PHF6*	*PHOX2B*	*PIGA*	*PIK3C2B **	*PIK3CA*
*PIK3CB*	*PIK3CD*	*PIK3CG*	*PIK3R1*	*PIK3R2*	*PIM1*	*PLAG1 **	*PLCG1*	*PLCG2*	*PML **
*PMS1*	*PMS2*	*POLD1 **	*POLE*	*POLR2A*	*POT1*	*PPARG **	*PPM1D*	*PPP2R1A*	*PRDM1*
*PREX2*	*PRKAR1A*	*PRKCA*	*PRKCI **	*PRKDC*	*PRKN*	*PRPF40B*	*PRPF8*	*PRSS8 **	*PTCH1*
*PTEN*	*PTPN11*	*PTPRD*	*PTPRT*	*QKI **	*RAB35 **	*RAC1*	*RAD21*	*RAD50*	*RAD51*
*RAD51B*	*RAD51C*	*RAD51D*	*RAF1*	*RANBP2 **	*RARA **	*RASA1*	*RB1*	*RBM10*	*RECQL **
*RECQL4 **	*REL **	*RELA **	*RET*	*RHOA*	*RICTOR*	*RINT1 **	*RIT1*	*RNF43*	*ROS1 **
*RPS15*	*RPS19*	*RPS20 **	*RPS6KA3*	*RPTOR*	*RRAGC*	*RSPO2 **	*RSPO3 **	*RUNX1*	*RUNX1T1 **
*SAMD9*	*SAMD9L*	*SBDS **	*SDHA*	*SDHAF2*	*SDHB*	*SDHC*	*SDHD*	*SETBP1*	*SETD2*
*SF1*	*SF3A1*	*SF3B1*	*SGK1*	*SH2B3*	*SIX1*	*SIX2*	*SLIT2 **	*SLX4*	*SMAD2*
*SMAD3*	*SMAD4*	*SMARCA4*	*SMARCB1*	*SMARCE1*	*SMC1A*	*SMC3*	*SMO*	*SNCAIP **	*SOCS1*
*SOS1*	*SOX10 **	*SOX2*	*SOX9 **	*SPEN*	*SPOP*	*SPTA1*	*SRC*	*SRP72*	*SRSF2*
*SS18 **	*STAG2*	*STAT3*	*STAT4 **	*STAT5B*	*STAT6*	*STK11*	*SUFU*	*SUZ12*	*SYK **
*TAF1*	*TBL1XR1*	*TBX3*	*TBXT **	*TCF12*	*TCF3*	*TCF7L2*	*TEK*	*TENT5C **	*TERC*
*TERT*	*TET1 **	*TET2*	*TFE3 **	*TFEB **	*TGFBR2*	*TMEM127*	*TMPRSS2 **	*TNFAIP3*	*TNFRSF14*
*TOP1*	*TOP2A **	*TP53*	*TP63*	*TPMT **	*TRAF3*	*TRAF7*	*TRRAP*	*TSC1*	*TSC2*
*TSHR*	*TYK2*	*U2AF1*	*U2AF2*	*USP6 **	*USP7*	*VAV1*	*VEGFA*	*VHL*	*WRN*
*WT1*	*XPC **	*XPO1*	*XRCC2 **	*YAP1 **	*YWHAE **	*ZBTB2 **	*ZFTA **	*ZMYM3*	*ZNF217 **
*ZNF703 **	*ZNF750*	*ZRSR2*							
(**b**)									
*ABL1*	*ABL2*	*AKT3*	*ALK*	*ARHGAP26*	*AXL*	*BCL10*	*BCL11B*	*BCL2*	*BCL6*
*BCOR*	*BCORL1*	*BCR*	*BIRC3*	*BRAF*	*BRD3*	*BRD4*	*BTG1*	*C11ORF95*	*CAMTA1*
*CARD11*	*CBFB*	*CBL*	*CCND1*	*CCND3*	*CDK6*	*CHIC2*	*CIC*	*CIITA*	*COL1A1*
*CREBBP*	*CRLF2*	*CSF1R*	*CTNNB1 **	*DEK*	*DNAJB1*	*DUSP22*	*EBF1*	*EGFR ^*	*EP300*
*EPC1*	*EPOR*	*ERBB2*	*ERG*	*ETV1*	*ETV4*	*ETV5*	*ETV6*	*EWSR1*	*FER*
*FGFR1*	*FGFR2*	*FGFR3*	*FGR*	*FLT3*	*FOS*	*FOSB*	*FOXO1*	*FOXP1*	*FOXR2*
*FUS*	*GLI1*	*GLI2*	*GLIS2*	*HLF*	*HMGA2*	*HTRA1*	*IKZF1*	*IKZF2*	*IKZF3*
*IRF4*	*ITK*	*JAK1*	*JAK2*	*JAK3*	*JAZF1*	*KAT6A*	*KIF5B*	*KLF2*	*KMT2A*
*LMO1*	*LYN*	*MALT1*	*MAML2*	*MAST1*	*MAST2*	*MEAF6*	*MECOM*	*MEF2D*	*MET $*
*MKL1*	*MKL2*	*MLLT10*	*MLLT4*	*MN1*	*MSMB*	*MYB*	*MYBL1*	*MYC*	*MYH11*
*MYH9*	*NCOA1*	*NCOA2*	*NCOA3*	*NF1*	*NF2*	*NFKB2*	*NOTCH1*	*NOTCH2*	*NPM1*
*NR4A3*	*NRG1*	*NSD1*	*NTRK1*	*NTRK2*	*NTRK3*	*NUMBL*	*NUP214*	*NUP98*	*NUTM1*
*PAG1*	*PAX3*	*PAX5*	*PAX7*	*PAX8*	*PDCD1LG2*	*PDGFB*	*PDGFRA*	*PDGFRB*	*PHF1*
*PIK3CA*	*PKN1*	*PLAG1*	*PML*	*PPARG*	*PRDM1*	*PRDM16*	*PRKACA*	*PRKCA*	*PRKCB*
*PTCH1*	*PTK2B*	*PVT1*	*QKI*	*RAF1*	*RARA*	*RELA*	*RET*	*ROS1*	*RSPO2*
*RSPO3*	*RUNX1*	*RUNX1T1*	*SETD2*	*SS18*	*SSX1*	*SSX2*	*SSX4*	*STAT6*	*SUZ12*
*SYK*	*TAF15*	*TAL1*	*TBL1XR1*	*TCF12*	*TCF3*	*TERT*	*TET1*	*TFE3*	*TFEB*
*TFG*	*THADA*	*TMPRSS2*	*TOP1*	*TP63*	*TYK2*	*USP6*	*VGLL2*	*WHSC1*	*YAP1*
*YWHAE*	*ZCCHC7*	*ZNF384*							
